# Facilitators of and Barriers to Teachers’ Engagement With Consumer Technologies for Stress Management: Qualitative Study

**DOI:** 10.2196/50457

**Published:** 2024-10-22

**Authors:** Julia B Manning, Ann Blandford, Julian Edbrooke-Childs

**Affiliations:** 1 UCL Interaction Centre Department of Computer Science University College London London United Kingdom; 2 UCL Institute of Healthcare Engineering University College London London United Kingdom; 3 Evidence-based Practice Unit University College London and Anna Freud Centre London United Kingdom

**Keywords:** teachers, stress, workplace, self-management, digital health, technology, qualitative, context, high schools, wearables, apps, human-computer interaction, HCI, personal informatics

## Abstract

**Background:**

Consumer technology is increasingly being adopted to support personal stress management, including by teachers. Multidisciplinary research has contributed some knowledge of design and features that can help detect and manage workplace stress. However, there is less understanding of what facilitates engagement with ubiquitous “off the shelf” technologies, particularly in a specific occupational setting. An understanding of features that facilitate or inhibit technology use, and the influences of contexts on the manner of interaction, could improve teachers’ stress-management opportunities.

**Objective:**

The aim of the study was to investigate the interaction features that facilitated or inhibited engagement with 4 consumer technologies chosen by teachers for stress management, as well as the influence of the educational contexts on their engagement. We also examined how use of well-being technology could be better supported in the school.

**Methods:**

The choice of consumer technologies was categorized in a taxonomy for English secondary school teachers according to stress-management strategies and digital features. Due to the COVID-19 pandemic, we adapted the study so that working from home in the summer could be contrasted with being back in school. Thus, a longitudinal study intended for 6 weeks in the summer term (in 2020) was extended into the autumn term, lasting up to 27 weeks. Teachers chose to use either a Withings smartwatch or Wysa, Daylio, or Teacher Tapp apps. Two semistructured interviews and web-based surveys were conducted with 8 teachers in England in the summer term, and 6 (75%) of them took part in a third interview in the autumn term. Interviews were analyzed using reflexive thematic analysis informed by interpretive phenomenological analysis.

**Results:**

Technology elements and characteristics such as passive data collation, brevity of interaction, discreet appearance, reminders, and data visualization were described by teachers as facilitators. Lack of instructions and information on features, connectivity, extended interaction requirements, and nondifferentiation of activity and exercise data were described as barriers. Mesocontextual barriers to engagement were also reported, particularly when teachers were back on school premises, including temporal constraints, social stigma, and lack of private space to de-stress. Teachers had ideas for feature improvements and how educational leadership normalizing teachers’ stress management with consumer technologies could benefit the school culture.

**Conclusions:**

Having preselected their stress-management strategies, teachers were able to harness design features to support themselves over an extended period. There could be an important role for digital interventions as part of teachers’ stress management, which the school leadership would need to leverage to maximize their potential. The findings add to the holistic understanding of situated self-care and should inform developers’ considerations for occupational digital stress support.

## Introduction

### Background

Workplace stress was reported nearly 200 years ago and has been described by Jackson [1] as “psychological pressures generated by the unfettered growth of industrial and technological capitalism.” When these pressures overwhelm, they cause distress (hereafter just referred to as stress) and stress continues as a pervasive phenomenon today. More than twice as many people report stress, depression, or anxiety causing them to miss work compared with any other reason, with teachers demonstrating nearly double the incidence rates of the general population [2]. Stress is a determinant of well-being and can be defined as “the adverse reaction people have to excessive pressures or other types of demand placed on them” [3]. Persistent, excessive stress is a risk factor for illness, including affective disorders such as anxiety and depression [4] and cardiovascular disease [5]; the latter is associated with burnout in even young female teachers [6]. Therefore, prevention or early intervention to reduce the effects of stress remains of huge importance.

Stress management interventions have proliferated, motivated by challenges including therapy costs, accessibility, and unmet needs of employees [7-11] with over 1000 stress apps found in web-based stores [12]. Increasingly, research shows digital tools can also be harnessed to detect, reduce, and manage stress. These technologies have used a variety of methods, including approaches broadly classified as personal informatics [13] that involve gathering self-report or automatic sensor data from everyday life to support reflection [14-16]. Other approaches include condition-based education [17], self-management through guided therapy [18,19], peer or social support [20,21], and gaming or entertainment [22,23].

Digital platforms mean that personal stress management interventions are now accessible throughout the working day via web- and computer-based programs [24], apps [25-28], and wearables [29,30]. In addition, popular websites have been repurposed to provide stress management interventions [10].

People’s ability to access stress management interventions is also highly reliant on their contexts. Methods such as contextual inquiry explore how technology fits into everyday life and tend to consider the context of the organization or sector [31,32]. Also important are the contextual influences on the ability to engage with digital interventions such as social norms, culture, time, and location [33]. Human-computer interaction analysis only occasionally extends to a distal understanding of the value chain in how interventions work in context and why [34]. Yet, this is important for people using stress management interventions in the workplace, both with novel designs [35-40] and consumer devices [41-44], as such an understanding of contextual influences on interaction with features would inform evaluation, iteration, and use. Such real-world studies are few, especially outside office environments.

There have been some multidisciplinary reviews of digital workplace interventions, yielding little insight into influences and features, but stress is usually grouped with other conditions in the continuum of mental health and well-being, and few studies are situated in the public sector [45-48]. Many studies in these reviews focus on novel system design or the evaluation of a single digital solution. Less is known about facilitators for and barriers to employees using consumer technologies to manage occupational stress, despite the proliferation of commercial tracking tools and the consequent amenability of studying their real-world use [16,49].

### Aims

We know that many teachers already have their own stress management strategies [50,51] and some are already using consumer technologies to support them [52]. This study took a phenomenological approach to understand how digital technology could facilitate teachers’ stress management in their real-world, complex layered contexts of the school setting [53]. Our research questions were as follows:

1. What were the facilitators and barriers to teachers engaging with their technology?
2. How could the school support the use of consumer technology for stress self-management? Coincidentally, this study commenced as the global COVID-19 pandemic took hold. Most teachers were suddenly working remotely from home at first, so we had the opportunity to compare facilitators and barriers to technology use between the home and school environments.

In this paper, we first describe digital detection and management of workplace stress and then review what is known about facilitators and barriers to interaction with such digital tools. We then present our study, describing which technologies were used by the teachers and presenting the research findings. We discuss what features teachers found to help them with stress management and what were the impediments to self-care. Finally, we consider what this means for school leadership in the context of education.

### Related Work

#### Stress Detection

Digital tools can enable both self-report and automatic detection of stress. Manually, this can be achieved through ecological momentary assessment [54], and popular consumer apps such as Daylio and Moodkit (Thriveport LLC) allow reflection on captured feelings or symptoms associated with stress and well-being [49,55]. Automatic detection can be through wearable sensors using galvanic skin response (GSR), also known as electrodermal activity [43], through photoplethysmography (PPG) to detect heart rate variation [30,56], and from smartphone text language [57]. Equivalence of stress detection in the real world has been demonstrated between manual self-reports and both automated GSR or voice-based analysis [58]. Heart rate and heart rate variation are a burgeoning area of research [59,60]. Automatic sensor data enable reflection on both stress-associated heart rate directly and on associated stress variables such as mood, activity, and sleep [16,60-63]. Caution is required for stress detection, both because detection itself can be a stressor [36] and due to discrepancy in perception between automated reports and self-evaluation, both in terms of not only severity [64] but also lack of differentiation between distress and eustress (the latter being a positive motivator for growth, development, and change) [4,65].

By definition, employees’ stress is strongly linked to the contexts in which they work. Some workplace studies have illustrated occupational associations—for instance, a study of desk-based office workers’ changes in the use of a mouse or keyboard showed it to be a valid proxy for stress [66]; in quiet environments, smartphone sensors have also detected stress through the human voice [58,67]; and phone use data are also correlated with stress [68]. However, these examples also show their contextual sensitivity and irrelevance for teachers in their noisy, dynamic, and didactic environments. Few studies have examined stress detection among teachers. Fitbits (Google Fitbit) using PPG to capture heart rate data were used to help teachers pinpoint stressors [69] and Philips DTI-2 devices (Philips) using GSR were worn by vocational school teachers to explore associations between data and stressors [70]. Research on the use of stress-tracking (personal informatics) technologies in other real-world contexts is sparse [16,41], although it is of increasing interest as machine learning is applied to sensor data, enabling prediction as well as detection [35,71].

Given that “context” is used to describe everything from high-level surroundings to individual-level characteristics, we drew on multidisciplinary domains of education, human factor, and informatics literature [72-75] to define the education workplace as the macrocontext; define the temporal, physical, social, and cultural factors as the mesocontexts; and define the individual’s personally chosen strategy as the microcontext.

#### Stress Management in the Workplace

Detecting stress and understanding that contexts influence experiences of stress is one side of the equation. Assisting people to then manage this stress is the other side, and critical to this is not only the digital medium or concept but what features are appropriate for the context. For the purposes of this paper, features are regarded as mechanisms of interaction or engagement, including reflection, therapy, and social or professional support [33,76,77] with facilitating elements such as information, notifications, or passive data collection and characteristics such as the medium and esthetics [78]. We have little information on what appropriate features are for school teachers, although there may be some relevant insight from other workplaces. Some studies have reported that participants would recommend the intervention to a friend but did not give any reasons as to why or when [29]. They also omitted key information, such as whether interventions were accessed at work, particularly in busy contexts, such as health care [79-81].

#### Facilitating Features

Personal data collection has facilitated correlations between stress and real-world stressors [49], including for teachers from their wearable data [69,70], and enabled anticipation of stress for mitigation planning among students [82]. One population-specific stress intervention, a role-playing game, showed design could be relatable to a specific culture [83], although role-playing games for stress management generally appear to be untested [84].

Timing and choice of stress intervention or prompts have given indicators of user receptivity. Desk-based workers preferred prompts at the start of a task or day, during lunch breaks, or during the postlunch circadian rhythm dip of 2 PM to 3 PM [38]. Autonomy to choose interventions has been linked to enhanced motivation and adherence to mental health interventions [85]. Where cognitive behavioral therapy (CBT), mindfulness, and tailored stress management therapies have been delivered digitally to employees from a variety of occupations, they have fared better in the workplace when supported or guided than when not supported or guided [46,85,86].

In education, despite some extensive trials with teachers [50,69,70,87-91], qualitative insights on facilitating features of digital stress interventions are few. However, temporal flexibility of intervention [20,91], informational support [92], and anonymity and peer social support [20] have been credited as facilitating features. In other workplaces, interesting content and interactivity, short completion time, progress tracking for reflection and reminders [93], and personalization in apps were appreciated [8,94]; and flexibility, goals, and calming strategies were desired [95]. Synthesizing this workplace literature, facilitating themes for digitally supported stress management emerge as a personal choice over timing, supported reflection, and relevance.

#### Barriers to Use and Interaction

Barriers to digital workplace stress interventions are from heterogeneous sources but include concepts relevant to teaching. They can include a lack of information: not understanding how to respond to data or lack of instructions and the need for “communities of practice” [41,70,96,97]. Mesocontextual barriers have included time pressures [95,98,99], lack of privacy [97] and cultural stigma from having to admit a need or the intervention being for mental health,’ or lack of active management support for intervention deployment [93,97,100,101]. Personal factors include being unmotivated [93] or lacking autonomy or choice over design interventions [100,102].

## Methods

### Overview

The goal of this study was to investigate how consumer technologies could facilitate stress management in the school environment.

A longitudinal study was planned that used semistructured interviews, as well as open and closed-question surveys. Before the first interview, workshops were conducted to help teachers make an informed choice of consumer technology based on understanding their personal stress management strategy and technology preferences. Consumer technologies were framed as digital companions when presented to teachers in workshops. This framing drew on the framing of apps as “life companions” by Klasnja and Pratt [103], acknowledging users’ autonomy [104], emphasizing the mediating and collaborative role of the technology [105], and avoiding any commercial connotations of simply being a consumer Referred to by name or simply as technologies in this paper, those chosen by teachers for this study were the Withings Steel HR smartwatch (that uses PPG to detect heart rate changes); Wysa (Wysa Ltd), an artificial intelligence chatbot based on CBT techniques; Daylio, a mood diary and activity tracking app; and Teacher Tapp, a daily teachers survey with an educational blog. Support was given to the teachers in acquiring their choice of technology from those populating a rigorously compiled taxonomy based on suitability, availability, evaluation, security, validity, and cost; the design of which is described elsewhere [106]. Reasons for teachers’ choices are described in detail in a companion paper on their experiences but include apparent ease of use, to inform stress correlations; enable passive collation of data; and give insight into sleeping patterns [107].

The interview data were supplemented with feedback questionnaires, memos and note taking, and email responses (including from nonparticipants, with their permission). The details, rationale, and elements of these methods are now described.

The orientation of this research was experiential qualitative research [108]. The approach was not guided by an existing theory but combined inductive (bottom-up from the data), cognitive, and social constructivist (the participants’ viewpoints) reflexive thematic analysis [109,110]. The constructivist paradigm is familiar in both human-computer interaction and education research, thus enabling epistemic consistency.

### Teacher Recruitment

The initial selection of participants was purposive. The inclusion criteria were to be a head of year or assistant head with pastoral responsibilities. Willingness to both talk about stress and to trial their chosen technology to support their stress management strategy; and not currently receiving treatment for a clinical stress disorder such as posttraumatic stress disorder or acute stress disorders were specified in the information sheet. The latter was stipulated to avoid interference between this study and potential prescribed interventions. No prior training in stress interventions was required.

Recruitment was from schools in one multi-academy trust (MAT) based in South London. The Trust had been approached for several reasons. First, MATs have a single governance structure, providing some reduction of internal organizational variation, but each has its own leadership team. Second, the MAT had 3 secondary schools in the South London region whose principals were willing to participate, providing a geographic locus and city context for the research. While there are always some differences in the local housing and labor markets, the good transport links, a predominance of business and service sector jobs along with low unemployment, gave some cohesion to area characteristics [111]. Third, the MAT had a track record of interest in staff well-being. By working in the same city with one MAT and teachers with the same role (head of year), the aim was to ensure some contextual consistency.

Of the 16 teachers who originally volunteered to take part before the pandemic (having been invited by the MAT directors and principals), 8 (50%) were still able to participate after the COVID-19 pandemic precipitated a national lockdown, with participation now facilitated on the web. None were previously known to the researcher. Four (N=16, 25%) of the teachers who dropped out gave reasons and consented to have them noted. These included extra childcare responsibilities, their partner working for the National Health Service and being overwhelmed, and an increase in teaching load. The other 4 did not communicate their reasons.

### Ethical Considerations

This research was approved by the University College London Research Ethics Committee (UCLIC_1920_004_Staff_ BlandfordManning). Information and consent forms were sent electronically to teachers before the workshops, followed up with reminder emails if they had not been received by the day before the workshop. The consent and information forms specified the purpose of the workshop and subsequent interviews and survey should they choose to take part, as well as the interviewer’s (JBM) department and university, and that the data would contribute to a final study being undertaken as part of her PhD. The data collected were anonymized, with personal names, identifying remarks, or identifiable topics mentioned in the interview audio recordings removed from the transcripts. Participation was voluntary, but a flat participation fee of £150 (US $183.10) was paid to cover the costs of the participant obtaining their chosen technology. Participants had the right to withdraw from the study at any time.

Due to the pandemic, only 2 participants spent more time in school than working from home during the summer leg of the study. Six (75%) of the 8 teachers extended their involvement from the “intended” study into the autumn term back in school. The 2 (25%) who did not continue gave no reason, but there was still a huge pressure on schools at that time. This autumn interview is described as the “extended” period in the results.

### Workshops

The choice of consumer technology was facilitated by the presentation of a taxonomy of stress management interventions [106]. The workshop was piloted and refined with 3 teachers not involved in the study. Workshops used Zoom (Zoom Video Communications), PowerPoint (Microsoft), and Mentimeter and were arranged at a convenient time for the teachers. Three teachers attended the first workshop, 2 the second, and 3 the third. Two people ran the workshops, a teacher and the researcher (JBM), who is an experienced clinician and policy researcher interested in professionals’ stress.

The workshops were designed to first introduce the study concept. Second, they were designed to allow teachers the time and information to understand the different ways in which they consciously or subconsciously seek to manage stress. This was intended to facilitate the identification of personal stress management strategies. Third, the workshops were designed to introduce teachers to the different ways in which technology could conceptually support their stress management strategies. Finally, they were designed to describe each of the technology options open to them for the forthcoming study. Mentimeter, an interactive presentation software, was adopted to enable anonymous crowdsourcing of ideas from teachers. The software aggregates responses to questions and displays them in real time as a word cloud.

Data were gathered both through feedback forms and from the workshop recordings. Transcripts were made of the latter, but the data were mainly reflections on understanding stress and contextual information relating to COVID-19 pandemic changes. The feedback forms allowed teachers to record their choice of technology, along with why they chose it, and when and where they anticipated using it. Participants were reminded only to submit their feedback form if they wished to proceed. All participants returned their forms. The feedback form also captured some demographic data and contextual feedback, largely related to changes brought about by the pandemic. These data along with the comments from the workshop allowed for some tailoring of the semistructured interviews according to technology choice and the altered teaching arrangements due to the COVID-19 pandemic.

After receiving the feedback form, the researcher contacted the participant to arrange (1) payment of the flat participation fee and (2) schedule the time of their first interview. The staggered timings of the workshops meant that some teachers had their technology ahead of others. One teacher had considered trying an online CBT program but on examination reported it as too medical. Another had hoped to use the Fit2Teach Pro app but at the time, technology purchases needed to be made and the app had not been updated within the previous 6 months, so it was excluded as an option. Another teacher wanted to try both the Withings watch and Wysa app, but as the cost exceeded the flat participation fee, she opted for the Withings watch only (Table 1).

**Table 1:** Teachers’ technology choices and summary findings.

Teacher number	Technology choice	Time as teacher (years)	Chosen stress management strategy and technology-supported concept (first or second)	Duration of technology use (from download or receipt and nighttime use)	Extra features desired (or already present but undiscovered)
T1	Daylio and Wysa apps	>10	Cognitive and reflection; cognitive and self-management	20 weeks continuous then a few missed days before the autumn half term	Better data visualization Congratulatory messaging
T2	Withings watch—wearable	>10	Physiological and reflection or peer-to-peer social	22 weeks (including nights)	Ability to link raised heart rate with correlations in a diary Raised heart rate warning
T3	Withings watch—wearable	>20	Physiological and reflection or peer-to-peer social	22 weeks continuous (nightwear quickly discontinued)	Ability to track gardening or not just sports activity
T4	Withings watch-—wearable	<10	Physiological and reflection or peer-to-peer social	15 weeks continuous then 4× per week for the next 7 weeks (nightwear quickly discontinued)	Link between calorie expenditure and nutritional advice Slimmer-too bulky to wear at night Naming alarms
T5	Teacher Tapp	>10	Educational and reflection	7 weeks 2 days continuous, then occasional use for 7 weeks in the autumn term	Mindfulness section and link to a yoga workout as a reminder to take breaks
T6	Withings watch—wearable	>20	Physiological and reflection or peer-to-peer social	27 weeks continuous (including nights) included planned health leave for 6 weeks	Longer-range Bluetooth connectivity More than 3 alarms Naming alarms
T7	Withings watch—wearable	>10	Physiological and reflection or peer-to-peer social	7 weeks of intermittent wear; also picked up Teacher Tapp intermittently	None
T8	Withings watch—wearable	<10	Physiological and reflection or peer-to-peer social	8 weeks continuous	Indicators of normal parameters with focus on solutions or signposting in the Withings app (eg, raised heart rate warning)

### Semistructured Interview Details

Eight teachers took part in 2 one-to-one interviews planned for the intended study; 6 (75%) teachers went on to participate in a further interview for the extended study in the autumn. At each interview, an overview of the study was first recapped and the teacher’s current circumstances were discussed. Open-ended questions were then framed around the teacher’s personal choice of technology followed by probing of contexts (eg, physical, social, and cultural). Questions tailored to the technology allowed in-depth explanations of the facilitation of and barriers to the use of features and their ideas for improvement. After the interview, each participant’s responses were copied into a file and added to a pseudonymized case study folder for that participant in NVivo (Lumivero). The length of each interview ranged from 32 to 76 minutes.

### Open and Closed Survey Questions

In the intended study, spanning 6 to 7 weeks of the summer term, surveys were sent after about 4 weeks to prompt thinking about activity, data gathered, frequency of use, and stress symptoms. Three surveys were set up using Microsoft Forms, and each participant was sent a link by email and staggered through the week. The questions were arranged around themes and included branching from responses to ensure subsequent questions were logical and related to their technology choice. Closed yes or no, multiple-choice, Likert scale, and open responses were included. The response rate varied from 100% (8/8, first and last day of the week) to 75% (6/8, midweek). The answers informed the second and third interview questions.

In the extended study, 14 closed questions were asked at the end of the interview as a way of capturing final participant thoughts on technology features and the influence of their occupational contexts on use. These were simple agree-disagree responses that allowed for greater confidence in the analysis. All survey responses also provided more data for triangulation.

### Data Analysis

All interview recordings were transcribed verbatim and identifiers were removed. Transcripts were not returned to participants, as (1) the researcher did not want to add a time burden to the teachers taking part, (2) the evidence suggests that doing so adds little to the accuracy of the transcript [112], and (3) this retains the responses precisely as they had been given at the time of the interview when the teacher had the time and space to respond naturally. Transcribed interviews were checked against the audio recordings for accuracy.

Reflexive thematic analysis was undertaken of the whole data set (transcripts, surveys, researchers notes, and emails) [113] and informed by interpretive phenomenological analysis. NVivo (version 12) and SimpleMind (versions 1.22 and 1.31) software were used for the organization and development of codes, categories, relationships, and themes. Codes were shared with the coauthors (AB and JE-C), and a codebook was generated. In line with the advice of Braun and Clarke [110], a self-reflexive approach was taken throughout the research, including creating a rolling memo of reflections from the time of the interviews until the end of the study. These notes were shared with the coauthors during the stages of thematic analysis. Table 1 summarizes the teachers’ technology choice and supporting concepts, duration of use, strategy and mechanisms, and extra features desired.

## Results

### Overview

A total of 8 teachers completed the summer interviews, and 6 (75%) were involved in the extended study in the autumn of 2020. Most teachers were working from home during the summer term due to COVID-19 pandemic restrictions. They were all back in school in the autumn but in “bubbles” segregated by year group, moving between classes instead of students moving around the school and with extended school days due to staggered student arrival times. The sample was ethnically diverse, with just 1 male participant in both summer and autumn studies.

Teachers described how they began to use their technologies and which features facilitated their interaction with the technology and awareness of their stress. Quick and discreet interactions were complemented by passive data collection, making use simple, convenient, and private. Automated prompts and data collation facilitated easy engagement and awareness of stress patterns and associations. However, the teachers described having to be persistent with adoption, as inadequate instructions required them to experiment and use social media to discover more features. There was a particular issue reported with the Withings watch measuring sleep when they were awake and a desire that this wearable could automatically detect activity related to their job or their stress-relieving exercise. For fuller use and benefits to be realized, teachers described creating a culture in which it is socially acceptable to manage and talk about stress, where privacy was facilitated and self-care with technology was normal and personalized from the start of a teacher’s career. This would legitimize experiences of pressure and permit self-care.

The participant code is included at the end of quotations in the form of a teacher number (T1, T2, etc); the technology; and the term (summer or autumn) from which the quotation was taken.

### Theme 1: Technology Design Facilitated Interaction for Teachers

This theme captured how the design and features supported rapid technology use. Teachers conveyed a sense of confidence that interaction could therefore take place without anyone knowing.

#### Subtheme 1.1: Passive Collection of Data Is Convenient

Although the Withings watch can be set to record activities, it also automatically registers when the wearer goes for a walk. Teachers appreciated this convenience and the option to either reflect on data in the moment or at a time of their choosing. Teachers using the Withings watch liked the fact that it was “just there” on their wrist, and this in itself could motivate activity:

I go from here to work and walk or I do a walk, it will still register when you download it as a walk. Or you can then programme it and it will still register it as a walk. So that’s good in the fact that it knows it’s not just general walking around, it does it as an activity even though I haven’t put it and logged it as an activity.

T3, Withings, summer

In-the-moment reflection was facilitated by setting haptic notifications; one teacher described being able to set an alert triggered by her heart rate:

I know if I’m getting totally stressed, I’ll have a look at [the watch] now. And I’ll go, hold on, it’s not worth this, just slow down... you can set an alarm when your heart rate goes over a certain amount... Just a buzz, and it tells you where your reading is.

T6, Withings, autumn

#### Subtheme 1.2: Brevity of Interaction Simplifies Usability

Several teachers spoke not only of the convenience of passive data collection but also of this being complemented by the brevity with which data could be viewed. This cognitive ease of viewing the Withings watch was true for 2 apps as well, even though the data collection design required active engagement.

The contrast between working from home and in school can be seen in a teacher’s report of using her app in the summer and then toward the end of the autumn half term, when even the brief interaction could be overwhelmed by ongoing workplace demands:

As soon as [I] turn my phone [on] you see all the faces that come up straight away... And [later] I hear the brrrrng...I look down...I just do it quickly.

T1, Daylio, summer

But I know that I’ve missed days, not from not wanting to do it, but I forgot to do it. I’m using it then I say okay, I’ll do it in a minute.... I think the last two weeks, I’ve been really tired, really, really tired.

T1, Daylio, autumn

Another contrast could be seen between apps that offer tracking or treatment. While both were accessible, only the former was usable in school, although the understanding facilitated by the latter was appreciated:

I like Wysa. But.... You can’t just do a quick fix.... I was using Wysa, and it must have been about 45 minutes later [I thought] I’ve got to go to bed!...as a result of that...I don’t use Wysa as much.

T1, Wysa, summer

#### Subtheme 1.3: Discreet Appearance Encourages Use

The concept of cognitive ease also included the ability for discreet use, including using the messaging feature of the Withings watch to triage notifications, with the sender and start of message view enabling teachers to decide whether the whole message or email needed immediate attention or not. The subtle design of both the wearable and tracking app was commented on by most participants:

I’m not typing in anything. It’s very simple to use.... You can actually press the buttons and then press okay and then it just goes away straight away. Nobody needs to know why you are using [it] or what that’s for.

T1, Daylio, summer

The brevity of interaction or unobtrusive haptic notification built into the Withings watch worked together with discreet design to facilitate use:

I know now if my arm’s vibrating, I can just take a quick look. And if I think it is something that I need to address, then I can, and if it’s not, just ignore it. I got into trouble for having my phone out before.

T2, Withings, autumn

### Theme 2: Data Presentation Enabled Teachers’ Stress Awareness

#### Overview

The essence of the first theme was cognitive ease, and this was also true for data presentation. Use was further described in terms of how prompts and patterns made interacting with data simple. Teachers reported their data were a useful measure of quantification of their stress and they could see information relevant to educators.

#### Subtheme 2.1: Automated Reminder for Cognition or Reflection

As teachers used their technologies, both automation of questions linked to data collation and seeing raw data with the chance to capture other activities were appreciated as prompts to take further action. For the teachers using Daylio and Teacher Tapp, the automated reminders to record their emotional state or answer education-oriented survey questions facilitated the opportunity to stop and think, although the frequency of doing this was significantly curtailed when they were back in school full time. Some attributed this entirely to the ongoing pandemic:

Looking at it, and then it’ll prompt you, if you like, so how many more steps do you want to do, what have you done? It congratulates you, it just does nice stuff on it.

T6, Withings, summer

Teacher Tapp was asking you your thoughts, or...there were a couple of times it asked you specific stress questions as well...that made me think, okay, what am I doing? Should I try some mindfulness? Should I take some time off? Am I reflecting on my work?... During COVID and being [out of] school, [I used Teacher Tapp] almost 99–100%. Now that school has started, completely the opposite, I’d say I’ve probably used it ten times...if I was back to school and it wasn’t a pandemic, I’m still adamant I would use it.

T5, Teacher Tapp, autumn

#### Subtheme 2.2: Correlating Patterns in the Macrocontext of Education

Throughout the study, many teachers mentioned the patterns they could see in their data as being informative and relevant. This could simply be the visual display of their activity readings or tracking display, as shown in the Withing’s Health Mate companion app or on the Daylio app. It could be data showing a raised heart rate and making post hoc correlations between it and a stressful situation during the day or episodes of a particular in-school activity:

I’m looking at the data like steps, heart rate, because you can track it during the day when you go onto the app, and you can see your heart rate throughout the day. You can see where things, you can say, yes, that was where I was...dealing with a stressful situation.

T6, Withings, autumn

As the Daylio app allowed the tracking through emoticons, the teacher using it was able to see the connections between a less satisfactory day and her physical well-being:

I actually started to note the days that I’m not feeling physically well are the days, and I note it as a “meh” day, so you can see where the pattern is when I do that.

T1, Daylio, summer

Awareness of these patterns in the data was interlinked with the theme of relating data to fellow teachers.

#### Subtheme 2.3: Relevance of Information to Situation

Teachers described the data not only quantifying their stress but also facilitating education about their colleagues’ stress as well. Two described Teacher Tapp survey results as providing relevant data on the broader educational system. The teacher using the chatbot described how the interactions equipped her with the language to talk to fellow staff and this was an unanticipated benefit:

What Wysa has helped me with is managing other people because of all the tools that it gives you. So, even today, somebody was feeling a bit down.... And I said I’m sorry that you feel like that. It’s something that Wysa would say!... Wysa is very educational, it’s been more of an educational tool of helping me to understand.

T1, Wysa, autumn

### Theme 3: Barriers With Instructions, Accuracy, and Other Limitations

#### Overview

Contrasting with the facilitating features of design, barriers were first evident from descriptions of the designers’ apparent assumptions that consumer technologies use could be entirely intuitive. This created some friction in their use. The usefulness of the technology was hindered by a lack of information, with some participants believing that features were not available when they were and vice versa. Barriers also included a lack of automatic data differentiation and connectivity limitations. Once again, these subthemes are interconnected.

#### Subtheme 3.1: Inadequate Information Supplied on Technology Adoption

Five out of 6 teachers who used the Withings wearable commented on the lack of information provided with the watch. They all looked for more information on YouTube. Some teachers had found a feature that others thought was missing, but there was a strong sense that clearer instructions for all the technologies would have been appreciated:

I think for me, doing this has been more of a trial and error, and working out, oh this is what you do. But if there was a better instruction manual, it’d be easier to work through things.... The YouTube things are great because I’ll watch [and] they can [show you how to] do this and that.

T1, Daylio, summer

The inadequacy of instruction was related not only to the functional use of the Withings watch but also to what data constituted “normal” for the user and how data could vary. Some teachers were worried by their raised heart rate or commented that seeing their personal data and variation in isolation was worrying:

Also, the only thing with the watch and its limitations is, you don’t really have a national comparison.... As in, I find there’s ten beats per minute difference between me at the weekend, and me at school. So, on the weekend I average 73, 74 resting heart rate. Whereas in school, it’s 83, 84. And I’m like, is that normal?...Obviously, I’m more stressed, you’d expect that, but is that the same average (for other) people?

T8, Withings, summer

#### Subtheme 3.2: Insufficient Instructions on Technology Features and Limited Strategy Options

Alternative stress management features, narrowness of existing features, and lack of signposting were described by teachers as insufficiencies in their technology. The desire for a greater plurality of features was often related to alternative stress management strategies that teachers described when on the school premises. Instructional insufficiency was seen when one teacher, asked about improving the Withings watch, said that she would like there to be a food diary. However, this was already a feature on the companion app that another teacher had mentioned. Another example was where 2 teachers described the haptic notification function and how they would appreciate being able to set it to alert if their heart rate was raised, yet another teacher described that she had done this. However, the teacher who had managed this also expressed disappointment that the alarms she had set up as reminders to drink could not be named, yet other teachers had described naming their alarms:

What would I like the watch to be able to do?... Set multiple alarms more than I’m doing now, I think. You can set multiple alarms [but I would want to name] an alarm to say, have a water break.... I don’t set it for anything else [now] other than [as a] cue to have a drink, make sure I’m drinking.

T6, Withings, autumn

A point was also made that having exercise limited to sport was a missed opportunity to label data on activities, such as gardening or cleaning. Sometimes secondary technology concepts supported alternative stress management strategies, such as social connection, unanticipated before the social isolation of lockdown. However, some of the teachers wanted more strategies supported by their technology:

Maybe Teacher Tapp could be changed.... I just found as someone that likes to read and get information it...brought me into that mindset that I was still working.... I’d definitely make a part where there’s yoga or mindfulness sections allowing people to get away, just a different strategy.

T5, Teacher Tapp, autumn

#### Subtheme 3.3: Variation in Usefulness, Accuracy, and Accessibility

Comments on the accuracy of the data collected were mostly positive, with 1 participant who had previously used another activity tracker stating that she “couldn’t cheat” with the Withings watch, as she had been able to increase her activity reading simply through shaking her old tracker. However, inaccuracies were mentioned by several teachers, including the Withings watch recording them as sleeping when they were not:

One thing that I was confused about.... It measured me sleeping when I actually wasn’t.

T7, Withings, summer

Accessibility of the technology was a theme that meant different things to teachers. Teachers with the wearable reported appreciating that the watch was “just there” on their wrists and easy to view during the working day, but one needed spectacles to see it. Using a smartphone app during the school day required forward planning for proximity, either to simply access the app or for the short-range Bluetooth required for message transmission between the phone and watch:

Normally I used to just put [my phone] in my bag. But what I’ve done, I’ve bought a pouch... so if I’m wearing a dress or it’s something that hasn’t got pockets, then I can put it in my pouch and keep it on me.

T1, Daylio, summer

I haven’t got the connection [in school] to [receive messages]... You have to be within a certain distance, and I’m never within that distance...maybe... if I put my phone in my laptop bag...so that it’s with me, but the kids don’t know I’ve got it. Because I don’t like to have my phone around.

T6, Withings, autumn

Most teachers liked the presentation of data, but it was observed that a more visual display of data could be more compelling and relatable to the associated activity:

It [Daylio] gives you all the data, it breaks it down, say, how many times you’ve done this, how many times you’ve done this, etc...but I guess it would be good to have a visual...where you have a bar chart or something that’s blatantly obvious... a sunshine, or something, I don’t know. Feel good. Happy. Oh, wow, there’s that sunshine, that rainbow, wow...So, you’ve had these good days because you’ve done X, Y, Z.

T1, Daylio, autumn

#### Subtheme 3.4: Need for Differentiation for Data Relating to Stress Management Strategy

Descriptions of how the data were relevant to participants’ working lives as teachers included capturing the demands of the day. However, for some, differentiation between stress self-management activity from activity intrinsic to the job required remembering to activate specific tracking as it was not automatic. One teacher described it as an additional task, although possibly unrealistic, as 5 (83%) out of 6 teachers described a decrease in interaction with technologies overall in the autumn term:

I think for me...[the technology] was a really useful reminder for me to keep fit, to keep active...I think that really made a big difference to my mental wellbeing in the lockdown...[but] there’s no breakdown on where that activity is coming from in the day...I need to get better at...turn[ing] the activity on...to differentiate how much of my activity is actually coming from something good, and then how much of it is from walking around.

T4, Withings, autumn

The decrease in interaction in the autumn relates to the final theme of managing technology use in the mesocontexts of school life.

### Theme 4: Managing Mesocontextual Barriers Could Offer a Framework for a Caring School Culture

#### Overview

Barriers to engagement were also external to the technology. All the teachers using their technology in the physical school environment expressed a clear sense of competition between technology interaction and occupational demands. In addition, the ongoing pandemic meant that routines were not the same and the extra or different demands on teachers’ time sometimes eclipsed the use of the technology. Some wondered how their interactions would have been different had they begun using their technology while on school premises. Despite the mesocontextual barriers of time, space, and social and cultural norms, teachers’ raised awareness of stress management through technology notifications or data meant some did still try to activate stress management strategies during the school day.

Teachers considered that by leveraging personal technologies, schools could embed self-care from the start of a teacher’s career. The aim would be a school culture and environment in which self-management of stress became normative.

#### Subtheme 4.1: Competing Workplace Demands Can Overwhelm Use

Teachers described a frenetic working environment in school in the autumn, which had not been the case during lockdown and working from home. Four (66%) teachers talked about responding to technology alerts or notifications on the school premises; 3 of whom described this being done “on the run” between activities—if they responded at all. This was despite participant unanimity on the data-facilitated opportunity to reflect on personal well-being. This temporal barrier to interaction was possibly more noticeable due to teachers experiencing the contrast of job demands between working from home and working back in school:

I feel motivated when it beeps to tell me that I’ve done so many thousands of steps in the day, but I’m running around like a headless chicken so much that it [the technology] could be ringing or [alerting] emails or texts, and I don’t even notice it.

T2, Withings, autumn

Teachers still used their technology but most interacted with it less when back on school premises, some putting this down to the ongoing disrupting effects of COVID-19. Wysa was described as needing a “quiet space,” and both Wysa and Teacher Tapp needed time and “work” for interaction:

But because there’s a pandemic and things arise, you don’t have the routine...The [Teacher Tapp] alarm goes off at 3.30 but I’m always caught in the middle of something...with people getting sent home or staggered departures or parents wanting to meet you, it just fell behind.

T5, Teacher Tapp, autumn

#### Subtheme 4.2: A Physical Environment That Facilitates Taking a Break and Privacy

Staff talked about the need to have a brief respite from being in public view or always “performing” but having no staff room to facilitate this. To gain just a few minutes’ peace, 5 (63%) out of 8 teachers in the summer described that before the pandemic they had hid in the toilets, in the comer of a classroom, or in a colleague’s office or gone outdoors where they could not be found. One teacher said there was no point in trying to have a break during the school day as she would always be disturbed. Now that they wanted to use their technology or act on the data to de-stress, they described being more aware of the physical barriers to doing so. Others talked about making an extra effort in the autumn to act on the knowledge gained from their technology in the summer when there were few physical constraints:

I think things that I learnt over the lockdown, and with the technology, I definitely have brought into this term.... [I am] trying to take a lunch break.... I’m just trying to take at least 15 minutes of the day to just sit down because I wasn’t even sitting down, really, before. Ever.

T4, Withings, autumn

For one teacher, teaching in a new environment having moved school in the autumn, the added structures in place meant that having a break was more realistic:

I think just better structures, so different things happening at the different times that you know about and everybody just sticks to what they’re supposed to do...But break, lunch, I am on duty sometimes but I make sure I’m supporting the staff. But yes, to get a break, again, that’s down to structure and routine so I’m more able to get a break [now].

T5, Teacher Tapp, autumn

#### Subtheme 4.3: Making It Socially Acceptable to Talk About Stress

From the start of the study, teachers had reported a reluctance to talk about stress in school or to share sentiments of stress with some of their colleagues:

I think teachers’ stress is still quite taboo, to be honest, within the workplace.... I think it can be seen as a sign of weakness.

T4, Withings, summer

Having used their technology, some teachers considered that data could be used to demonstrate objectively to the leadership where situations in school were more stressful. If leadership encouraged discussion of stress based on data this could facilitate both technology use and targeted problem-solving:

I think if you could show them the value of using it, [say] with the tool, these [data show] where I’m most stressed, this is where I’m not... if you put me in here this is where I stress. ...if we could show that there’s ways of tracking stress, I don’t know, they might value it.

T6, Withings, autumn

#### Subtheme 4.4: Normalizing and Personalizing Self-Care With Technology From the Start

Some participants described technologies as an opportunity to introduce self-care right from the start of the profession. If the school leadership facilitated this it would send the cultural message from the outset that teachers’ self-care is important:

Yes...I think that we should start to use them [technologies] earlier on in [our] teacher career, because then it helps you to focus on your wellbeing as an individual, and help you with your stress, your work/life balance.... As a teacher, you don’t actually look after you, you look after everybody else’s people. But if you can’t look after yourself, it’s going to be very difficult for you to look after others.

T1, Dalyio and Wysa, autumn

Some considered that using technology for self-care would also remind teachers that they had a responsibility to look after themselves, not that they bore the entire burden of stress management but that they had an important part to play:

I think there’s a lot to be done, and I think technology is certainly a way that we could manage that, because I think it’s very easy, and I recognise this in myself big time...to blame the job, blame the role, blame SLT, blame the marking, blame the kids, which obviously is a huge factor. But I think actually there’s a lot more we could do as individuals, just to take that time to dedicate to ourselves, reduce our stress levels in whatever way that might be.

T4, Withings, summer

## Discussion

### Principal Findings

To the best of our knowledge, this is the first study to describe facilitators and barriers to personally chosen technology support for stress self-management in the educational context, along with insight into how schools could facilitate such interventions. The findings complement those of teachers’ experiences, which demonstrate how a more holistic understanding of stress, and permission to manage it is generated as teachers found meaning in their data and interactions [107].

As shown in our findings, the themes generated indicate specific features, data collation, and technology-supported concepts that facilitated teachers’ use during their working day. In contrast, the barriers to use centered around a lack of information and signposting and external, school-related mesocontexts that inhibited use or putting their stress-relieving action into practice as planned. These themes are now unpacked further.

### Insight Into Design Facilitators of Technology Use by Teachers

Teachers expressed their appreciation of the wearable appearing to be a watch, not an obvious tracking device. They also liked the rapidity with which Withings watch data could be viewed and the Daylio app could be used, thus preventing potentially stigmatizing exposure. These themes reflect the persistent taboo of experiencing stress, and teachers’ fear of not being perceived to be able to cope. Discreet “design” has been noted previously as a facilitator for recording feelings [49,114] and for the use of technologies by employees for stress management [93,115]. It has also been observed that the esthetic of design of wearables is important [116]. Added to the knowledge that short interventions are important for stress management for providers and users [95], including in constrained workplaces [98], we suggest designers should ensure that where the perceived risk of stigma in the workplace remains, design camouflage would encourage engagement for teachers.

Immediacy is increasingly being facilitated by wearables and sensors [117]. The Withings watch enabled stress awareness with cognitive ease both through passively collated data and through haptic notifications. Haptic stimulation has been shown to have a positive influence on attitude to accompanying messages and such a physical cue is motivational [118]. Our findings suggest that for teachers in their polychronically demanding environments, such a physical cue facilitates engagement up to a point, dependent on the individual and competing demands. The exception to immediacy was Wysa, which required deliberate, sustained engagement. Temporal constraints precluded use in school, but Wysa (the chatbot) was described as a “best friend” for giving contextually applicable advice that was both educational and transferable. Such high-effort, problem-solving interventions have been found to be more effective for IT workers than low-effort, positive distractions [8]. Therefore, we suggest that cognitive ease can also enable digitally supported stress management by virtue of ease of application of learning, not just the immediacy of information.

This applicability of the chatbot advice also links to the finding of the importance of contextually mediated reflection on patterns for engagement. This aligns with research hinting that more contextualization facilitates engagement [119] and the noted lack of contextualization in mood-tracking apps used by the general population [49]. A combination of data patterns that could be correlated by the teacher to their circumstances, and the legitimacy of the intervention as facilitated by the school leadership, enabled teachers to reflect on their stress associations and plan mitigating actions.

Teachers identified automated prompts for taking more steps or meditating, or for messages of affirmation when an activity had been undertaken, as helpful. This complements teachers’ reports of appreciating congratulatory text messages of support via WhatsApp [120]. Elsewhere, police officers using wearables to track activity [121] and working-age women with technologies meeting self-set goals [122] have also reported the motivational aspect of digital messages of encouragement. Prompts or reminders for stress management can be preplanned or automated through algorithmic analysis of data [35,82]. Conversely, when prompts are not received, stress awareness among Chinese adults was reduced as was their ability to manage their stress [41]. Even for people in less-restricted roles than teaching, receptivity to automated supportive messaging is highly dependent on contextual variables [123]. Future research could consider how messaging could be further enhanced to enhance stress management among employees in temporally demanding roles.

### Barriers to Technology Use Described by the Teachers

This study validated the established need for good data visualization with improvements suggested for the Daylio app and readability of the Withings watch’s digital display screen. Stress data visualization has been tested at the organizational level [39], but this would be difficult to translate to schools. The paucity of setup information supplied with the Withings watch was commented on by 5 (83%) out of the 6 users. Teachers resorted to YouTube (not the Withings website) for guidance, although not knowing what constituted “normal” data remained a problem for 2 of them. Social sensemaking with personal informatics has demonstrated that value can be gained from contextualizing data through comparison with peers [124], but the COVID-19 pandemic would have been a barrier to this. The app users also cited apparent design assumptions of intuitive use, particularly with Daylio, which delayed full adoption. These barriers to adoption extend the finding of inadequate technical instruction in a digital mental health review [125], adding a specific example of a wearable consumer technology adopted for stress management (out of 208 papers, only n=2, 0.01% were for wearables in the review by Borghouts et al [125], and these were for tracking serious mental illness). Wearable connectivity was found to be limited by Bluetooth range, a challenge noted generally with digital stress support [126], which for teachers specifically related to the proximity of their smartphone, often not kept with them [52]. Designers therefore need to remember that users still need comprehensive navigational advice and that they cannot assume proximity between wearables and smartphones for all occupations.

Some teachers were disappointed that the data indicated sleep when they were awake, despite Withings’ reputation for accuracy [127]. This has been noted in studies of Fitbit [61,128]. Liang et al [128] suggested the companion app should allow the user to edit their inaccurate data, an important consideration because inaccurate data is generally a reason for abandonment [129]. This remains a consideration as users perceive stress algorithms to be at odds with their subjective experiences [64].

No other inaccuracies were noted by teachers in this study. Several teachers wished that their technology offered alternative strategies, for example, relaxation as well as education, partly because stress symptoms can change over time. This adds to similar reports for a greater plurality of features or strategies from other studies [93,130-132]. However, this must be balanced against the extent of choice [133], as too much choice in digital tools could be overwhelming [134].

Temporal constraints led to reduced frequency of interaction with technology for most of the staff when back in school, a barrier to interaction that can only be discerned in real-world evaluations [135]. Notably, this was only detected because of the extraordinary circumstances of teachers able to compare working from home with working back in school and because sustained engagement was still demonstrated months after initiation. The text-heavy Wysa (never accessed in school) and Teacher Tapp (significantly reduced interaction in school) apps were the most affected. Counson et al [98] found that vocational intensity was a barrier to engagement with a stress management app cocreated with doctors and professional demands constrained time and enthusiasm for digital health interventions generally [136]. Thus, organization-level solutions are likely to be required as well, connecting to the final finding that teachers’ desire is for a workplace culture that facilitates self-care and caring. Such a culture requires the facilitation of mesocontexts, such as time and physical environment. Although other studies have used machine learning to judge the best timings for stress interventions for students and in offices [10,38,82,131], the polychronic nature of teachers’ work would make finding patterns in behavior and just-in-time interventions more challenging. In addition, during the school day, stress management can require privacy, for example, in the form of a staff room, with lack of privacy being a known deterrent to interaction generally for digital mental health [125].

### Implications for the School Workplace

The findings on both facilitators and barriers to teachers’ engagement suggest that school leadership could leverage technology to support stress management. Some alerts can prompt teachers’ just-in-time stress relief strategies or simple self-care such as having a drink. Yet, teachers also need supportive mesocontexts such as a private space in which to relax, protected time when they are undisturbed, and a culture in which stress is seen as a warning and not a weakness. This need for a positive organizational culture for stress support has been noted before by office workers considering the use of personal digital tracking and e-coaching [137]. As several teachers commented, what is the point of drinking water to keep hydrated, if there is no time or provision (in their building) or even acceptance that they will then need to use the bathroom.

### Strengths and Limitations

Rich, contextualized data on the adoption of and influences on technology use were collected over an extended period of up to 27 weeks, enabling deep and detailed insights into teachers’ engagement. All 8 heads of year completed the summer study and just 2 (25%) were lost to follow-up in the extended period. This longitudinal study provides evidence of the acceptability of consumer technology to teachers for stress management support and which features were of value for self-care in an occupation renowned for its stressfulness.

Taking part in the research inevitably meant there was some extrinsic motivation and would have raised awareness of stress and self-care; therefore, there could have been a digital placebo effect [138-140]. The pandemic led to the exceptional circumstances of both more attention being given to teachers’ well-being by the school leadership and the teachers beginning to use their technologies while working from home. This meant that all participants had time to adopt their technology in a quieter environment and could compare facilitators and barriers between the 2 settings. Further research could test digital stress support against an active control.

The choice of technology was framed by a specific taxonomy which enabled choice based on strategy and conceptualization of interaction. Given the pandemic, workshops were held on the web which prevented hands-on exploration of technologies before selection. However, the main author was available for queries throughout the study. Therefore, another limitation was that autonomy was limited to the technologies that populated the taxonomy, selected based on suitability, availability, usability, security, validity, and cost. In addition, there was no classification of technologies according to detection, management or prevention, just-in-time recovery, or resilience building. These concepts and characteristics could be tested to see if they assist in technology selection and self-care. Apart from Wysa, no technology was chosen that facilitated treatment or guided self-management nor was entertainment based. The reasons for this as well as encouraging a wider variety of interventions could be investigated further.

Another strength of this longitudinal study was that it was undertaken with teachers who taught many socioeconomically deprived students, typically a more stressful setting. Teachers in more affluent areas or working with more economically advantaged children are known to experience less stress [141-143]. Thus, the findings should benefit teachers in more challenging situations. It was poignant to hear teachers in the autumn term talking about children returning to school after the pandemic without even a pen, let alone lunch money.

The participant sample cannot be claimed to be representative of all secondary school heads of year, although this study makes an important contribution, given the dearth of research with middle managers. The sample was self-selecting and only one head of year was male, but the racial mix was diverse.

### Conclusions

Consumer technologies are quick, easy, and discreet to use; offer encouragement; and enable personalization. They could have an explicit role in facilitating teachers’ understanding and managing their stress. Developers should not make design assumptions of users intuitively discovering all features, and the ability to manually amend some data should be facilitated. School leadership has an important part to play in facilitating stress management, and technology use could contribute to a culture in which self-care is normative and discussions of stress are socially acceptable. The convenience and ease of technology engagement could be leveraged at the organizational level to integrate stress support. This invites the proposition that a teacher’s ability to act on technology data is a proxy measure for a caring school culture. This would require both protected time and space on the school premises. We hope our findings are of interest to policy makers and encourage developers of digital interventions to work with educators to ensure contextual insights inform the design of health and well-being technology.

## Abbreviations

CBT: cognitive behavioral therapy
GSR: galvanic skin response
MAT: multi-academy trust
PPG: photoplethysmography

## Data Availability

The data generated and analyzed during this study are restricted because the transcripts in their entirety could potentially identify participants and are confidential.
